# Supplementary material for: The causal relationship between the human gut microbiota and pyogenic arthritis: a Mendelian randomization study
Source: Front Cell Infect Microbiol. 2024 Nov 26;14:1452480. doi: 10.3389/fcimb.2024.1452480 (PMC11629706; doi:10.3389/fcimb.2024.1452480)
Supplement: Supplementary file 1 [file DataSheet1.zip › mendelian test/ebi-a-GCST90027501.csv_scatter.pdf]

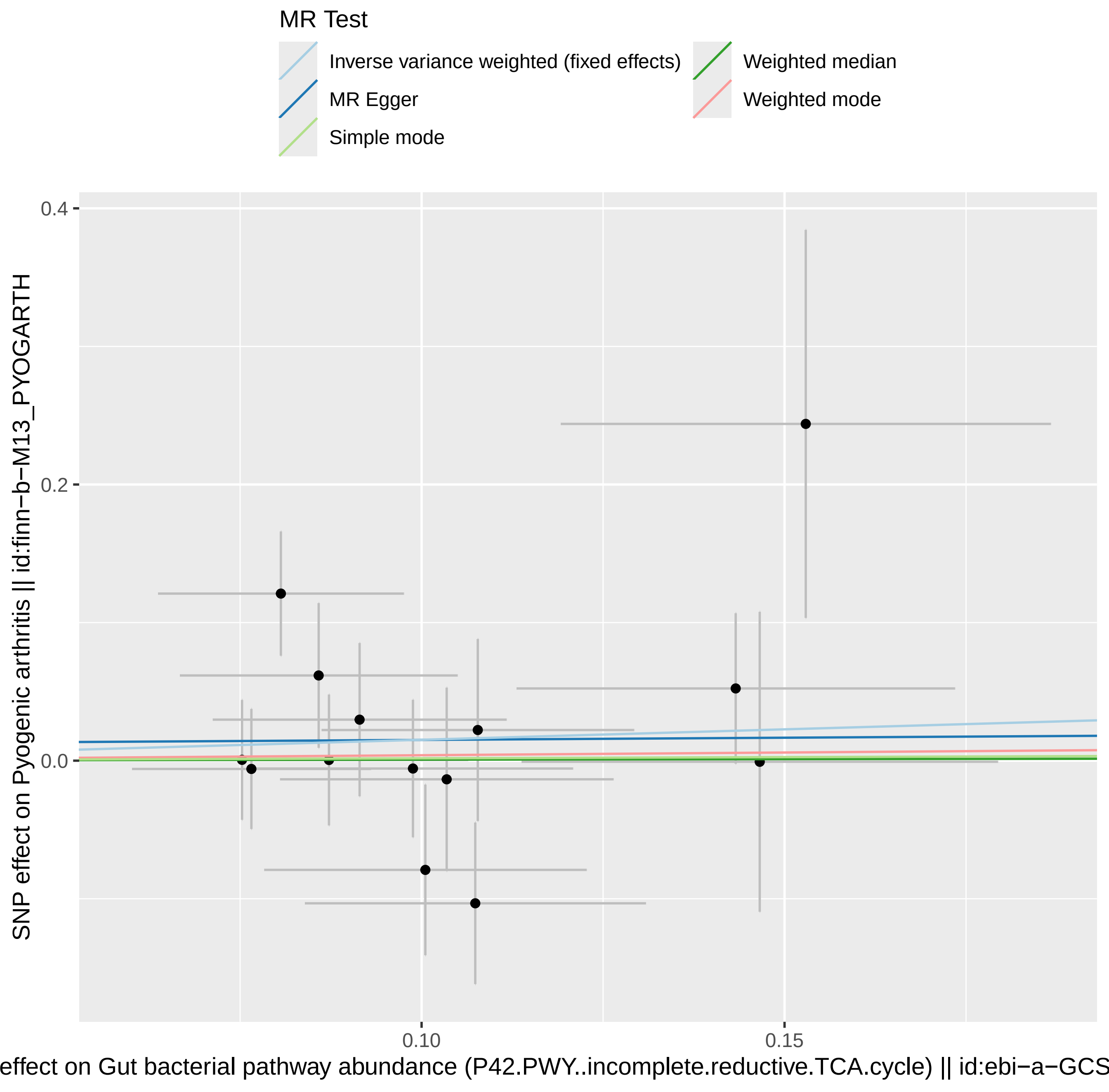

MR Test
Inverse variance weighted (fixed effects)
MR Egger
Simple mode
Weighted median
Weighted mode
0.4
0.2
0.0
0.10
0.15
SNP effect on Pyogenic arthritis || id:finn−b−M13_PYOGARTH
effect on Gut bacterial pathway abundance (P42.PWY..incomplete.reductive.TCA.cycle) || id:ebi−a−GCS
